# The Polyketide Components of Waxes and the *Cer-cqu* Gene Cluster Encoding a Novel Polyketide Synthase, the β-Diketone Synthase, DKS

**DOI:** 10.3390/plants6030028

**Published:** 2017-07-10

**Authors:** Penny von Wettstein-Knowles

**Affiliations:** Section for Biomolecular Sciences, Department of Biology, University of Copenhagen, Ole Maaloees Vej 5, DK-2200 Copenhagen N, Denmark; knowles@bio.ku.dk; Tel.: +45-3532-2180

**Keywords:** type III polyketide synthase (PKS), diketone synthase (DKS), β-diketones, hydroxy-β-diketones, esterified alkan-2-ols, *Cer-cqu* gene cluster, plant waxes, cytochrome P450, lipase/carboxyl esterase/thioesterase

## Abstract

The primary function of the outermost, lipophilic layer of plant aerial surfaces, called the cuticle, is preventing non-stomatal water loss. Its exterior surface is often decorated with wax crystals, imparting a blue–grey color. Identification of the barley *Cer-c*, *-q* and *-u* genes forming the 101 kb *Cer-cqu* gene cluster encoding a novel polyketide synthase—the β-diketone synthase (DKS), a lipase/carboxyl transferase, and a P450 hydroxylase, respectively, establishes a new, major pathway for the synthesis of plant waxes. The major product is a β-diketone (14,16-hentriacontane) aliphatic that forms long, thin crystalline tubes. A pathway branch leads to the formation of esterified alkan-2-ols.

## 1. Introduction 

Polyketide synthases (PKSs) are closely related to fatty acid synthase (FAS) enzyme complexes, but differ in failing to carry out one or more of the three reactions removing the 3-oxo (β-keto) group after each extension. Type III or chalcone synthase-like PKSs are homodimeric enzymes carrying out sequential condensations. All three reactions are thus omitted, thereby introducing oxo groups into the growing carbon skeleton. As few as one, and up to as many as eight sequential elongations can take place. These enzymes exhibit substrate specificity (normally for CoA linked molecules), chain elongation (using malonyl-CoA as a donor), and in most cases cyclization activities; all of which are attributable to the shape and size of the primer substrate binding pocket [1,2,3]. 

Three types of polyketides have been identified in plant epicuticular waxes: β-diketones, alkan-2-ol esters, and alkylresorcinols (ARs). Table 1 specifies the source of identified β-diketones, as well as their chain lengths, and positions of the oxo groups. Those from plant waxes have primarily 29, 31 and 33 carbon skeletons, with the positions of the oxo groups varying from 6,8 to 16,18. Most variable are those from sunflower, with not only acyl chains with a wide range of oxo groups, but also those including phenyl groups. A quite different series with oxo groups in only one position, very close to the end of the chain (2,4), are present in sphagnum, vanilla, and wheat. While they are prominent components of the neutral lipids in vanilla pod gum, they are present in trace amounts in subfossil sphagnum and wheat waxes. Alkan-2-ols with C_7–17_ odd chain lengths have been reported less frequently, and only once in the absence of β-diketones, on sorghum seedling leaves (Table 2). ARs are phenolic lipids, with alkyl side chains with varying degrees of unsaturation consisting of 13–29 carbons on carbon 5 of 1,3-dihydroxybenzene, that occur in minor amounts in Gramineae waxes, in a cuticle layer external to seed coats, as well as in root exudates [20].

Three β-ketoacyl-ACP synthase (KAS) enzymes participate in the reiterative reactions of plastidial FAS synthesizing fatty acids with KASII specializing in the final extension from 16 to 18. The 3-oxo group is removed after each extension, resulting in fully reduced, saturated acyl chains. One destination of the C_16_ and _18_ FAS products is the endoplasmic reticulum (ER), where related β-ketoacyl-CoA synthase (KCS) enzymes can continue elongation to at least 26 carbons, with elimination of the 3-oxo group in each cycle. The resulting acyl chains serve as precursors for the ubiquitous wax aliphatics, by functioning as substrates for associated enzyme systems, giving rise to aliphatics such as alkanes, primary alcohols and alkan-1-ol esters [21]. Additional functional groups can be introduced into the primary products of both the polyketide and KCS pathways; for example, a hydroxy group into the β-diketone skeleton to give a hydroxy-β-diketone, or into a primary alcohol to give a diol [22]. 

**Figure 1:**
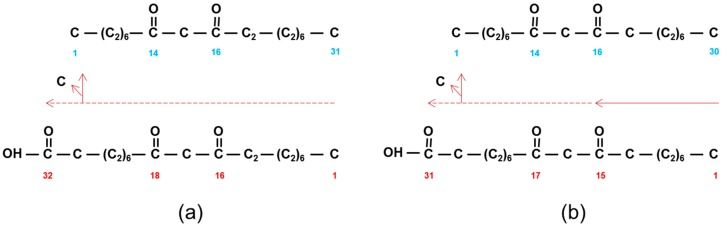
Structure and IUPAC (blue) nomenclature of β-diketones compared to their biosynthetic nomenclature (red). (**a**) Hentriacontane-14,16-dione (C_31_) is derived from 16,18-dioxodotriacontanoic acid by loss of a carbon. Horizontal dashed red arrow denotes direction of synthesis by successive additions of C_2_-units. (**b**) Triacontane-14,16-dione (C_30_) is similarly derived from 15,17-dioxohentriacontanoic acid. Solid red arrow denotes pentadecanoic acid (C_15_) that is extended by C_2_-units as indicated by dashed red arrow.

The phenotype of a plant cuticle is in part dependent on the presence of crystals. The *eceriferum* (*cer*) mutants interfere with the synthesis or transport to the apoplast surface of the compounds forming the crystals. The long thin, hollow tubes on the uppermost leaf sheaths and exposed internodes, plus the glumes and lemmas of barley spikes, are attributable to the dominating polyketides, namely the β-diketones, of which 96% is hentriacontane (C_31_)-14,16-dione (Figure 1a) plus its 25-hydroxy derivative. They give rise to the blue–grey glaucous color. The organ specific distribution of these polyketides in other grasses differs, however, as illustrated in Table 3. In addition to polyketides, all barley waxes contain ubiquitous aliphatics derived from the KCS elongation system. Only such aliphatics are found on all barley leaf blades which are covered by small lobed plates attributable to dominating amounts of primary alcohols resulting in a dull green glaucosity. 

**Figure 2:**
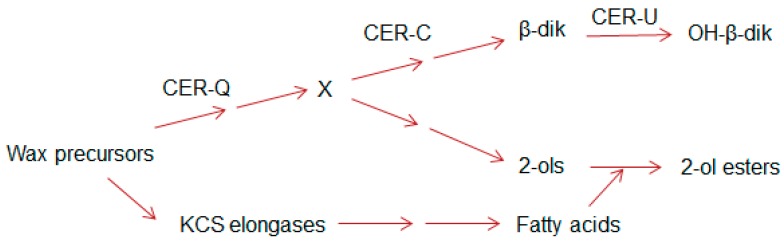
Sites of action of the *Cer-c*, *-q* and –*u* encoded proteins based on compositional analyses of the waxes of wild type and *cer* mutants with CER-U functioning as a hydroxylase; simplified from [16]. X denotes a common precursor for both the β-diketone aliphatics and the esterified alkan-2-ols. KCS elongases denote the elongation systems giving rise to the ubiquitous wax aliphatics.

In barley, more than 75 *cer* complementation groups have been identified that reduce glaucosity or result in bright green, non-glaucous surfaces [29]. The three with the most mutations, *Cer-c*, *-q*, and *-u* (with 202, 155, and 148, respectively) all affect stem and spike phenotypes [26,29]. Mutants of the first two are non-glaucous, and those of the third have a reduced glaucosity. Early chemical analyses of the waxes from the wild type and five of these mutants revealed that the *Cer-c*, *-q* and *-u* complementation groups affected only the β-diketone components of the wax, and that the function of *Cer-u* was to insert a hydroxyl group onto the C_31_-14,16-dione [30]. Continued exploitation of the *cer* mutants revealed that the presence of esterified alkan-2-ols was correlated with that of the β-diketones [15,16]. Moreover, while *cer-q* mutants impeded synthesis of both the esterified alkan-2-ols and β-diketones, *cer-c* mutants blocked only the latter, suggesting the biosynthetic relationship shown in Figure 2 (modified from [16]).

## 2. Discussion

### 2.1. Identifying the *Cer-c, -q* and -*u* Genes

In addition to the mutants noted above, 13 apparent multiple mutations all involving *Cer-c*, *-q* and/or *-u* among the barley *cer* mutant collection were identified. This observation led to an experiment to map them by looking for wild types produced by crossing over in a background of mutants. That none were obtained among the 26,933 gametes tested inferred that all three complementation groups were within 0.0012 mu of one another [31]. This was similar or less than the distances mapped at that time between alleles of the barley *li*, *ml*-*o* and *glx* loci. Early trisomic mapping experiments had localized all three *cer* genes to the end of a chromosome later shown to be 2H [32]. Combined these observations suggested that *Cer-c*, *-q* and *-u* formed either a tightly linked gene cluster, or a multifunctional gene [31]. That all pairwise combinations of *cer*-*c*, -*q* and *-u* mutants were present among the apparent multiples, inferred that not all could be attributed to deletions, even though five of the six were induced by neutrons implying deletion events. After 36 years, the question was finally resolved by exploiting the rapidly developing genomic resources and mapping populations in barley [26], and thereafter confirmed [27], as summarized below. 

### 2.2. The *Cer-cqu* Gene Cluster in Barley 

**Figure 3:**
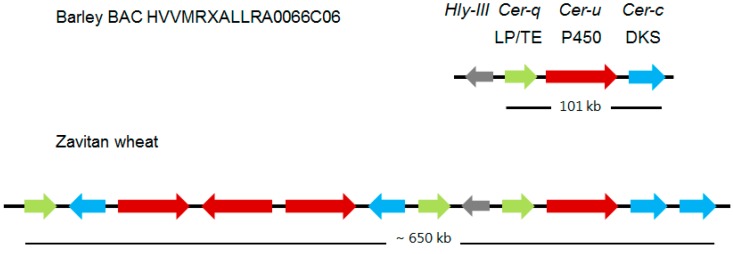
Comparison of the *Cer-cqu* gene clusters encoding wax β-diketone polyketides in barley and wheat. The latter cluster has undergone numerous duplication events since these species diverged. Only the 11 of the 15 Zavitan genes with potential function having homology to the *Cer-c* (blue), -*q* (green), and *-u* (red) genes of the barley cluster are shown. Those with highest homology to the barley genes are aligned vertically although the two DKS sequences at the right end of the wheat cluster cannot be discriminated between on this basis. Albeit associated with both clusters the *Hyl-lll* gene does not participate in polyketide biosynthesis. LP/TE, lipase/thioesterase; DKS, diketone synthase; P450, cytochrome P450 enzyme. Broad arrows represent relative lengths of the specified barley MLOCs and amount to only 5.3 kb of the 101 kb long cluster. Derived from [26] and [27].

Continued mapping experiments with the introduction of molecular markers revealed that *Cer-c* was situated in a subtelomeric region of chromosome arm 2HS [32]. Using the Bowman near-isogenic lines carrying *Cer-c*, *-q* and *–u* mutations, plus creating and analyzing appropriate mapping populations combined with putative functions for *Cer-c* and *-u* (see “Deducing the functions for Cer-U and -C in the wax polyketide pathway”) lead to identifying five potential candidates for these two genes. A candidate for *Cer*-*q* was also selected on the basis of its annotation and close proximity to the other five. Exploiting mutants with potential mutations in *Cer-c* plus *Cer*-*q* as well as *Cer-u*, six induced by neutrons and one by γ rays, revealed that three of the candidates were missing in six of the investigated lines. That the three candidates indeed encoded *Cer-c*, *-q*, and *-u*, was substantiated by sequencing more than 50 mutants distributed throughout each gene [26]. Tight linkage of the three genes was confirmed by identifying and sequencing the pertinent BAC. The three genes form a gene cluster extending over 101 kb, designated *Cer-cqu*, as shown in Figure 3, which discloses that the order is *Cer*-*q*, *-u*, and -*c*. This implies that the double mutant *cer-cq* was mistakenly classified [33], as confirmed by molecular analysis identifying a triple mutant [34]. Thus, only *cer-qu* and *-uc* double mutants occurred among the multiples. The intervening sequences on the BAC are filled with transposable elements, and the genes are highly expressed in flag leaf sheaths of wild type, as expected [26]. When the pertinent BAC has been integrated into the barley chromosome 2 map, whether or not additional genes belong to the cluster can be approached. If additional genes do belong, their mutation they will not affect glaucosity. 

### 2.3. A Much Larger *Cer-cqu* Gene Cluster Occurs in Wheat

As part of a study to understand the molecular nature of the wheat *W1* locus determining glaucousness of the uppermost leaf sheaths, peduncles, spikes plus abaxial flag leaf surfaces (Table 3), three highly expressed sets of genes located in the subterminal region of chromosome 2BS were identified as having homology to barley *Cer-c*, *-q*, or *-u;* 5, 4 and 6 genes, respectively, of which 4, 3 and 4 were neither pseudogenes, nor carried deletions [27] (Figure 3). The requisite duplications were deduced to occur after barley–wheat divergence. The barley stripe mosaic virus mediated gene silencing system was exploited to show that the pertinent, glaucous cuticle surfaces of the Bobwhite wheat cultivar became non-glaucous when the *Cer-c* and -*q* wheat orthologs were silenced [27]. These results demonstrated that one or more of the 4 *Cer-c* and 3 *Cer*-*q* wheat orthologs clustered in this region are indeed involved in synthesis of the β-diketone carbon skeleton. To confirm that one or more of the 4 *Cer-u* identified wheat orthologs is correct will require a similar silencing experiment in which the absence of hydroxy-β-diketones is confirmed by wax analyses, as glaucousness is not greatly affected, if at all, by the relative amounts of hydroxy-β-diketones to β-diketones [16]. Combining the presence of several potential homologues of each gene in the cluster with the ploidy of wheat, gives rise to the possibility that isomers with different substrate specificities exist. If true for wheat *Cer-q*, this could explain the bimodal distribution of the esterified alkan-2-ols with maximums at carbons 7 and 15 [19] (Table 2). 

**Figure 4:**
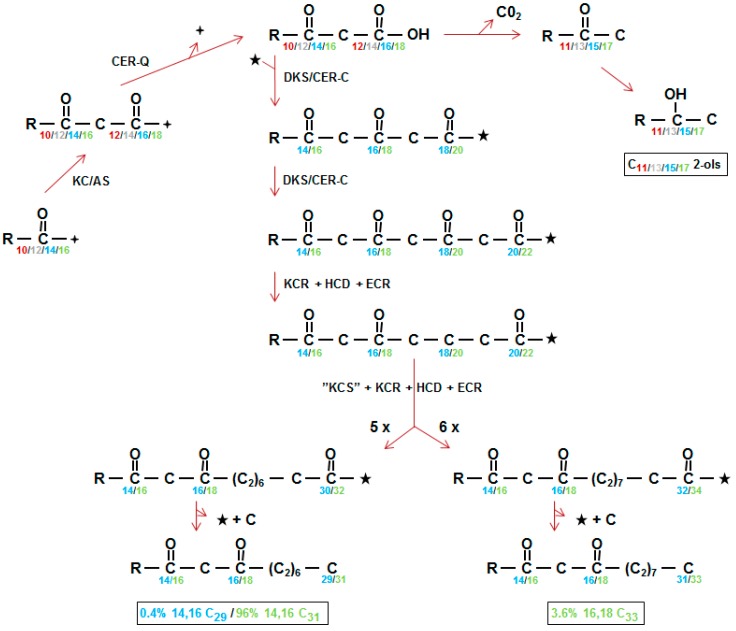
The polyketide pathway synthesizing β-diketones and alkan-2-ols. (Left, middle) KC/AS, either KCS, one of the β-ketoacyl-CoA synthases constructing carbon chains of the ubiquitous wax aliphatics in the endoplasmic reticulum or KAS, the β-ketoacyl-ACP synthase participating in fatty acid synthesis in plastids. CER-Q, a lipase/carboxyl transferase/thioesterase which is envisaged to hydrolyze the 3-oxo substrate (top, center) from ACP, CoA or lipid (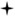). The 3-oxo substrate is then decarboxylated to form methylketones that are converted to alkan-2-ols (top right). Neither methylketones nor free alkan-2-ols occur in the wax, instead the latter are found as the alcohol moiety of esters (not shown). DKS/CER-C is the diketone synthase carrying out two extensions in barley giving a tetraketide intermediate (middle) presumably after activation of the 3-oxo substrate by CoA (★). KCR, β-ketoacyl-CoA reductase; HCD; hydroxyacyl-CoA dehydratase, ECR; enoyl-CoA reductase; “KCS”, condensing enzyme carrying out 5 or 6 extensions (5X, 6X) of the DKS synthesized tetraketides; C, carbon released by unknown mechanism. Numbering in direction of synthesis, except in boxes which give the IUPAC name requiring numbering in opposite direction of synthesis, of the three in vivo synthesized β-diketones [38]. Weight % of the β-diketones is given in Table 4. The homologs of CER-Q and DKS in wheat are named Diketone Metabolism-Hydrolase and Diketone Metabolism-PKS, DMH and DMP, respectively [27].

In wheat, the Inhibitor of wax 1 (*Iw1*) gene is a dominant suppressor of wax polyketides. The very recent cloning of this gene in durum wheat reveals that it encodes a miRNA whose primary transcript of 1051 bases forms a hairpin, because of an inverted repeat [35]. The latter has >80% homology to its 3 *Cer-q* wheat homologs that have been designated *W1-COE* [35] and *DMH* [27] (see Figure 4 text), and are the target of the predominant 21 nucleotide miRNA, miRW1, associated with the non-glaucous phenotype. An interesting question for the future is the significance of this regulation. 

### 2.4. Establishing the Barley Wax Polyketide Biosynthetic Pathway

Results of early radioactive acetate incorporation experiments using intact spikes, inferred that the 31 carbon β-diketone skeleton was synthesized by addition of C_2_ units from the C_31_- to the C_1_-end (Figure 1A in [36]). This approach was extended to include additional fatty acids as potential substrates, using tissue slices from spikes minus awns, as well as pretreatments with inhibitors potassium cyanide, sodium arsenite, 2-mercaptoethanol, and 1,4-dithiothreitol [5,37]. The shorter fatty acids lauric (C_12_), myristic (C_14_), and palmitic (C_16_), as well as C_16_-CoA, were excellent β-diketone precursors, but stearic acid (C_18_) as well as C_18_-CoA were not, although they served as precursors for the other KCS derived epicuticular aliphatics. Final confirmation of elongation and its direction were obtained by demonstrating that feeding [1^−14^C]-pentadecanoic (C_15_) fatty acid gave rise to a novel C_30_ β-diketone with the oxo groups on carbons 14 and 16, and label in the C_16–30_ end [5] (Figure 1b). The results moreover established that the oxo groups were incorporated into the growing carbon chain during elongation. Subsequently, very low integration of label from 3-hydroxy C_14, 16_ and _18_ fatty acids, as well 3-hydroxy C_16_-CoA, eliminated these compounds as potential precursors of the β-diketones. By comparison, 3-oxo-C_16_-CoA was very efficacious, and the conclusion drawn that this molecule was a substrate for the elongation system giving rise to the β-diketones (Figure 4 center). Analyses of all data revealed that while this compound was an efficient precursor in vitro, circa 96% of the β-diketones in vivo are derived from a 3-oxo-C_18_ compound [38]. 

### 2.5. Esterified Alkan-2-ols Originate from a Branch Near the Origin of the β-Diketone Biosynthetic Pathway

The closer biosynthetic relationship of the esterified alkan-2-ols to the β-diketones, than to any of the other identified wax aliphatics in barley, was deduced from their presence only in those wild type waxes containing the β-diketones, and from the frequent simultaneous loss or reduction of both aliphatics in waxes of *cer* mutants [16] (Figure 2). Employing selected *cer* mutants, tissue slices and variously radioactive CoA substrates revealed that incorporation of [9,10-^3^H]-3-oxo-C_16_-CoA was very efficient, yielding a distribution of labelled alkan-2-ol esters matching that of their in vivo weight distribution [38]. The results established that the alkan-2-ols and the β-diketones had a common precursor (Figure 4 top center). *Cer-c* mutations result in an increase in the proportion of the alkan-2-ol versus 1-ol esters; for examples, see [23,31,39]. This presumably results from blocking the first CER-C reaction leading to rechanneling of 3-oxo-acyl precursors to the esterified alkan-2-ols (Figure 4 top center). 

To ascertain how the 3-oxo-acyl-CoAs were potentially converted into alkan-2-ols appropriately labelled C_15_- and C_17_-2-ones, as well as C_15_-2-ols, were tested [38]. The efficiency of conversion intimated that both the methylketones and alkan-2-ols were alkan-2-ol ester precursors. Assays of crude extracts from the tissue slices revealed the presence of a thioesterase cleaving within 10 min 60–70% of the CoA from [1-^14^C]-3-oxo-C_16_-CoA to give a radiolabeled 3-oxo-C_16_ fatty acid. In addition, the extracts were shown to contain a very active decarboxylase, which formed CO_2_ and a C_15_-2-one from 98% of the labelled 3-oxo-C_16_-CoA, within two hours. In both assays, the labelled substrate was stable when boiled tissue was used, intimating the absence of significant spontaneous hydrolysis and decarboxylation. These results suggested that, in vivo, a thioesterase cleaves 3-oxo-acyl-CoAs, giving 3-oxo-acyl chains that are then decarboxylated to a methylketone, and thereafter reduced to an alkan-2-ol for esterification (Figure 4). A similar system for formation of C_11_ and C_13_ methylketones in tomatoes was subsequently established [40], with the genes *ShMKS1* and *ShMKS2* encoding the requisite decarboxylase and thioesterase, respectively. The established biochemical pathway in tomatoes, plus the biochemical studies in barley and the presence of nonan-2-ol esters in the absence of β-diketones in sorghum [17], support the contention that enzymatic reactions are required for methylketone and alkan-2-ol formation in wax polyketide biosynthesis. This is in accord with earlier observations in milk, yeast, and rat liver microsomes, showing that decarboxylation only occurred after treatment with heat or base [38]. On the other hand, both CoA hydrolysis and α-methylketone formation by decarboxylation have been attributed to the type III PKS, benzalacetone synthase (BAS) [41]. Much more recently, the suggestion has been made that a spontaneous decarboxylation of the 3-oxo-acyl intermediate may be contributing to or replacing the decarboxylase activity [27,40,41]. The presence of an esterase in the barley tissue was deduced from synthesis of labelled esters when [2-^3^H]-C_15_-2-ol served as substrate.

### 2.6. Deducing the Functions of CER-U and -C in the Wax Polyketide Pathway

A preliminary outline of the biosynthetic pathway giving rise to the β-diketones and esterified alkan-2-ols was presented 40 years ago (Figure 2) with CER-U functioning as a hydroxylase [16]. The latter was confirmed when the *Cer-u* gene was shown to encode a P450 enzyme [26,27] whose mutation results in the accumulation of β-diketones with a corresponding decrease in hydroxy-β-diketones [30]. 

Initially, the elongation system giving rise to the β-diketones was designated a β-ketoacyl elongase to distinguish it from the KCS elongase systems giving rise to the ubiquitous KCS derived wax aliphatics, and the oxo groups were envisaged as being protected, and thereby retained, during subsequent extensions [42]. By 1993, the β-diketones were recognized as polyketides [43], but only in 2012 was the suggestion made that the β-ketoacyl elongase complex was in fact a PKS and designated pkKCS [44]. With the recent isolation and characterization of the *Cer-c* gene as a chalcone synthase-like PKS [26,27], the pkKCS was renamed diketone synthase (DKS) to intimate the polyketide product it encoded [26]. Figure 4 center shows the two elongations DKS carries out to give the tetraketide intermediate with the two oxo groups retained on carbons 14 and 16 (blue) or 16 and 18 (green) in the direction of synthesis. At least 99% of the in vivo synthesized β-diketones in barley initiate from a 3-oxo-C_18_ precursor (Figure 4, green). Interestingly, the oxo groups present on the final β-diketone carbon skeleton are not introduced by DKS, but are part of the substrate used for its first reaction. DKS action results in the addition of two more oxo groups, forming an intermediate tetraketide, which are removed during subsequent elongations (Figure 4). Precedence for such occurs when BAS carries out one extension, and the oxo group in the product is that present in the 4-coumaroyl-CoA substrate. That DKS lacks the cyclization activity characteristic of type III PKSs has precedent in BAS, CUS and WtPKS1 [41,45]. Instead of cyclization, BAS, for example, hydrolyzes and then decarboxylates the extension product to give benzalacetone [41]. 

Very recently, bifunctional C_31_ ketols (14,16 and 16,14) were identified in wheat and the proposal made that the oxo and hydroxy groups were inserted during synthesis of the carbon skeleton [13]. That is, the hydroxy group was introduced during the last FAS elongation step when the 3-oxo group was reduced to a hydroxy, resulting in a ketol, rather than a dioxo substrate for the first DKS reaction. As the chain length distribution of the wheat β-diketones is almost identical to that of those in barley (Table 4 in [13]), and both species are closely related evolutionarily, the pathway in Figure 4 is likely to function in wheat. If so, then only the 14,16 ketol could be formed. On the other hand, if tautomerization of the β-oxo groups occurs, then both ketols are possible [46]. More likely, is another possibility mentioned, of reducing one or the other of the oxo groups in the finished C_31_ β-diketone, which gives rise to both ketols. 

### 2.7. Toward the Function of CER-Q

**Figure 5:**
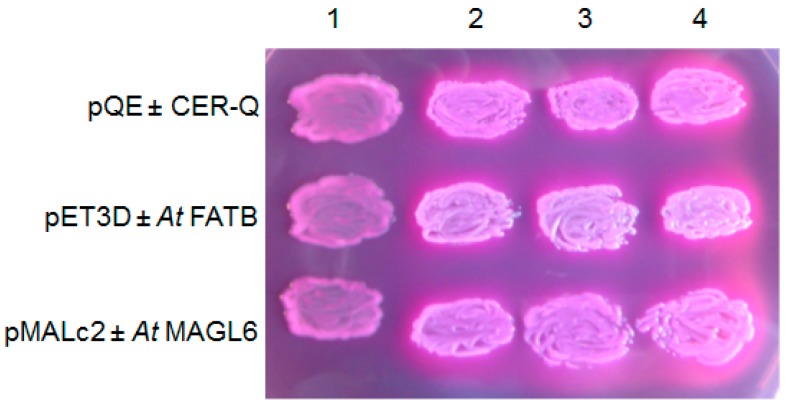
*Hv*CER-Q as the *At*FATB thioesterase, and *At*MAGL6 lipase expressed in *Escherichia* *coli* K27 (*fadD88*) on MacConkey plates excrete free acids into the medium that the pQE80, pET3D and pMAL-c2 vectors, respectively, do not. Lane 1, empty vectors; lanes 2, 3 and 4, vector plus designated insert in three independent transformants. Plates contained 0.5% lactose, 100 μg. mL^−1^ ampicillin, and 0.3 mM IPTG and was placed at 30 °C for 20 h.

CER-Q clearly functions upstream of both CER-C and -U (Figure 2). Isolation and annotation of the *Cer-q* gene [26,27] lead to its product being classified as a lipase/carboxyl transferase which is supported by structural modelling and analysis of the effect of the identified mutations in the α/β hydrolase core [26]. Combined with the biosynthetic studies summarized above, a function as a thioesterase hydrolase/lipase has been suggested for CER-Q. Two possibilities have been envisaged for its role in synthesis of mid-chain β-diketones, the class which most of the known ones belong to (Table 1); either to cleave 3-oxo intermediates from ACP during fatty acid synthesis in plastids, or to cleave them from a lipid in the cytoplasm [26,27,47]. That CER-Q is indeed capable of cleaving fatty acids is illustrated in Figure 5, where similarly to *At*FATB thioesterase and *At*MAGL6 lipase induced in the *fadD88* mutant *Escherichia coli* strain lacking acyl-CoA synthetase, the released fatty acids are excreted into the MacConkey medium. Cells doing so turn white and are encompassed by the red pigment in the MacConkey medium. Those not excreting fatty acids take up the pigment and become pink. [48,49]. To solve this question, barley CER-Q was expressed in *E. coli*, and cell extracts analyzed via gas chromatography-mass spectrometry [27]. A C_15_ methylketone was detected, and the deduction made that a 3-oxo-C_16_ acid was cleaved from fatty acid synthetase by CER-Q activity, that was then converted by one of two possibilities to the C_15_ methylketone. The 3-oxo-C_16_ acid is a precursor for the minor alkan-2-ols. It remains unclear why a C_17_ methylketone was not also detected when a 3-oxo-C_18_ acid represents the major precursor for the dominating β-diketones. This very preliminary experiment needs to be confirmed and expanded, analogous to the thorough characterization of the ALT thioesterases [50]. In this connection, the known substrate specificities of several thioesterases selecting short acyl chains is of interest, given the lengths of the esterified 2-ols (Table 2). That is, the insecticidal C_11_ and C_13_ methylketones of tomato trichomes arise from action of the thioesterase *Sh*MSK2, splitting ACP from 3-oxo-acyl-ACPs [40], to give 3-oxo-acyl acids. Moreover, four ALT thioesterases in *Arabidopsis* highly related to *Sh*MSK2 have been identified recently that choose among C_6–18_-ACPs as substrates [50]. One additional characterized plant acyl-ACP thioesterase capable of using intermediates of FAS, is FATB, whose isomers select among C_8–14_ chains [51]. Both the ALTs and FATB are, as *Sh*MSK2, plastidial. None of the specified thioesterases has homology to CER-Q.

Another system which deserves mention with respect to requiring short acyl chain precursors, is sporopollenin biosynthesis [52]. Sporopollenin monomers form a complex polymer, related to cutin and suberin, which is a primary component of the exine wall of pollen grains. A FATB-like thioesterase is thought to direct intermediate products of FAS to the cytoplasm. There, the *AtACOS5* encoding an acyl-CoA synthetase adds CoA to the C_12–18_ fatty acids, which are dispatched to the ER. Another hypothetical thioesterase enables these fatty acids to serve as substrates for the two PKSs *At*PKSA, and *At*PKSB, in the ER synthesizing tetraketide α-pyrones. These are the only yet known membrane localized PKSs. Where will DKS be localized?

**Figure 6:**
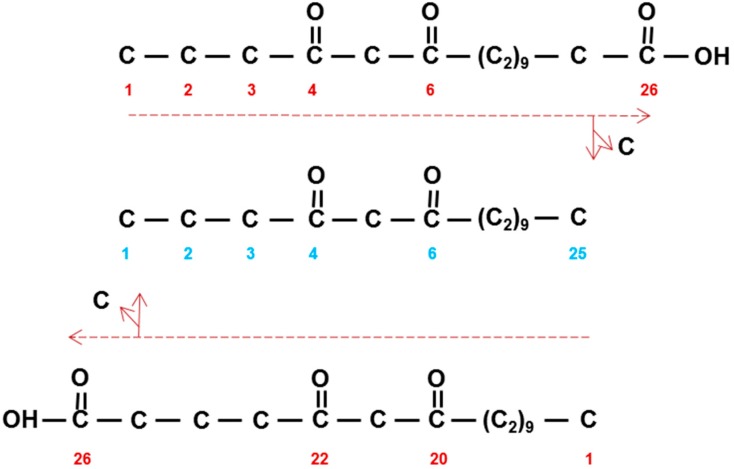
Nomenclature and possible synthesis of the carbon skeleton of pentacoasane-4,6-dione (middle). Synthesis (horizontal red arrows and numbers) in direction of (top) and in opposite direction of (bottom) IUPAC nomenclature (blue numbers). Vertical arrows show loss of carbon to give the C_25_ dione.

The above comparison of thioesterases using shorter acyl chains assumes that the numbering of the oxo groups in the β-diketones (Table 1) corresponds to that in the construction of the carbon skeletons. The other possibility is that the oxo groups are numbered in the opposite direction of synthesis. Only in barley have experiments established the direction of synthesis, namely that the 14,16-dioxo groups on the C_31_ skeleton corresponded to carbons 16 and 18 in synthesis (Figure 1 and Figure 4). An example from Table 1: 4,6-dioxo-C_25_ (Figure 6 middle) could be synthesized in direction of naming (Figure 6 top), or in the opposite direction (Figure 6 bottom), with the pertinent oxo groups introduced on carbons 20 and 22). If the latter is true, then long chain acyl-CoAs, rather than short chain acyl-ACPs, would be the substrates for CER-Q, which would split the acyl chains from CoA or lipids. If the 4,6-dioxo-C_25_ is indeed named in the opposite direction of synthesis, then these oxo groups represent the closest to the end of the acyl chain that they can occur, because the second DKS reaction results in a tetraketide β-diketone precursor that is not further extended (Figure 6 bottom). An identical mechanism cannot be invoked, however, in synthesis the of 2,4-dioxo-C_25–33_ diketones in vanilla bean pods, for example (Table 1). Only one DKS reaction is possible, giving a triketide (Figure 4 and Figure 6). Removal of the terminal carbon therefrom, would result in the 2,4-dioxo groups. But is this conceivable, as triketides are normally immediately extended by DKS? Yes, given that such a decarboxylation is an attribute of BAS [41]. If, however, synthesis is in the same direction as nomenclature, then the oxo groups are those present after the initial condensation in fatty acid synthesis, giving 3-oxo-butyric acid. Two additions thereto by a DKS, will give a C_8_ tetraketide. Thus, the mechanism of introduction of the two oxo groups on carbons 2 and 4 would be analogous to that for the other β-diketones in Table 1. Finally, 9–13 subsequent extensions would be required to give the final C_25–33_ carbon chains (see “What enzyme(s) extend the tetraketide formed by DKS?”). 

### 2.8. Why a *Cer-cqu* Cluster?

The primary function of cuticular wax is to prevent water loss, an attribute not previously attributed to gene clusters in plants [5]. Interestingly, no gene clusters encoding the ubiquitous wax aliphatics have been reported. One of the intriguing questions with respect to gene clusters for plant secondary metabolites, is their origin, which cannot be attributed to gene transfer from microbes [53]. While *Cer-q* certainly determines the first step in the DKS polyketide pathway (Figure 2 and Figure 4), and thus qualifies as a signature enzyme according to a recent definition [53], this term has also been used to define the enzyme evolved from primary metabolism, giving the unique metabolic structure to the clusters’ product [54]. In the latter case, this is *Cer-c*. Both are equally necessary for β-diketone synthesis, and rather than one recruiting the other to initiate the cluster, the random chance that both came to be closely linked might be a more logical way to envisage the origin of the *Cer-cqu* cluster. Blasting CER-C and -Q in Barlex identifies 76 and 78 additional annotated sequences, respectively. Of these, 9 CER-C and 14 CER-Q sequences occur on chromosome 2. The other chromosomes have a range of 4–13 and 3–19 additional annotated sequences, respectively. 

### 2.9. Besides Cer-c, -q and -u do Other Barley Cer Loci Function in the DKS Polyketide Pathway?

The *Cer-c*, *-q* and *-u* barley genes are peculiar to the DKS polyketide pathway which determines the predominating aliphatics in the wax coats on some cuticle surfaces. Distributions of the KCS derived aliphatics on these surfaces in their mutants are not modified, and in the case of *cer-u* and -*c*, neither are their wax loads. That of a *cer-q* mutant has not been determined [30]. Are there other analogous *Cer* loci affecting all cuticle surfaces producing β-diketone aliphatics? Potential candidates include *Cer*-*a*, -*b*, -*x*, -*z*, -*yl*, -*zl*, and -*yg*, with non-glaucous spike lemmas and glumes, plus uppermost internodes and leaf sheaths whose mutants have little, if any, of the β-diketone aliphatics analogous to those waxes of *cer-c* and -*q*. That the distributions of one or more KCS derived wax aliphatics are modified in some *cer*-*a*, -*b*, -*x* and -*yl* mutant spikes (see [55] for examples) implies that these genes most likely function before the DKS polyketide and KCS pathways diverge. A similar conclusion was drawn from studying 10 *cer*-*n* mutants in which no correlation was found between the extent of β-diketone reduction, and the effect on the alkane distributions [56]. The wide range in aliphatic distributions among the 10 *cer*-*n* mutants also emphasizes that deductions cannot be drawn based on the analysis of a single mutant whose KCS derived aliphatic distributions are not perturbed. Thus, nothing can be concluded about the roles of *cer*-*z* or -*zl* for which only one or no mutants, respectively, have been analyzed. On the other hand, the pleiotropic effects associated with all 12 mutants assigned to these two loci, infers a role outside the DKS polyketide pathway. Since mutations of the *Cer*-*yg* gene also result in non-glaucous leaves whose wax coats lack β-diketones, the function of *Cer*-*yg* is presumably also unrelated to the DKS polyketide pathway. 

In addition to CER-C, -Q and -U, at least a CoA synthetase is conceivably needed to form the substrate for the first DKS reaction as well as two enzymes being needed to remove the CoA and the carboxyl carbon from the fully elongated β-diketone carbon skeleton (Figure 4). That additional *Cer* genes have not been identified in the barley mutant collection raises the possibility that DKS, as BAS, carries out the latter two specified reactions [41]. Is it possible that DKS uses a 3-oxo-acyl substrate not linked to CoA, or does the enzyme participate in so many other reactions that its mutation would have far more drastic effects than on the polyketide pathway alone? Future studies will answer these questions. *Cer* genes for the alkan-2-ol branch of the polyketide pathway (thioesterase, decarboxylase and esterase, see above) will not be included in the mutant collection. Neither the methylketones nor the alkan-2-ols occur in the wax, and the esterified alkan-2-ols do not contribute to the cuticle phenotype. An esterase with homology to *Arabidopsis* wax ester synthase, has been identified close to the border of the wheat *Cer-cqu* cluster, and the suggestion made that this is potentially the enzyme esterifying the alkan-2-ols [27], but much work will be required to confirm this. 

### 2.10. What Enzyme(s) Extend the Tetraketide Formed by DKS?

In Figure 4, the enzyme(s) elongating the tetraketide product of the second DKS condensation is (are) designated “KCS”. Is it possible that the “KCS” are the same as the KCS carrying out the extensions of the equivalent chain lengths for the ubiquitous alkanes and primary alcohols? Two important facets must be kept in mind when contemplating this question. Firstly, the ubiquitous KCS elongation systems require *At*CER-22-like proteins for the final elongation steps [57]. When these genes are mutated, shorter chain lengths are present in the wax. Secondly, more than one third of the 1580 localized *cer* mutants in barley have been assigned to the *Cer-c*, *-q* and *-u* genes [29]. The probability is thus very unlikely that mutants of other *Cer* genes, unique to the DKS pathway, remain unidentified. This infers that either the answer to the posed question is “yes”, or DKS is responsible. If the former is true, then one would expect that an analogous shortening or absence of both the β-diketone and alkane carbon skeletons would occur simultaneously. Fifty-four *Cer* loci have been identified that reduce glaucousness of the spike lemmas and glumes, revealing that fewer β-diketone molecules are present. Isolating and characterizing the β-diketones from 32 mutants distributed among 26 *Cer* loci, however, gave essentially identical distributions for the C_29_, C_31_ and C_33_ chain lengths as characterizes wild type (Table 4). By contrast, analyzing the alkanes from 28 mutants of 22 *Cer* genes phenotypically classified as lacking wax, and hence β-diketones, revealed that while 15 had wild type alkane distributions, 13 exhibited a shift to shorter chain lengths, as exemplified by the data in Table 5. Combined, these data appear to rule out participation of *AtCER22*-*like* genes in the polyketide pathway. 

**Figure 7:**
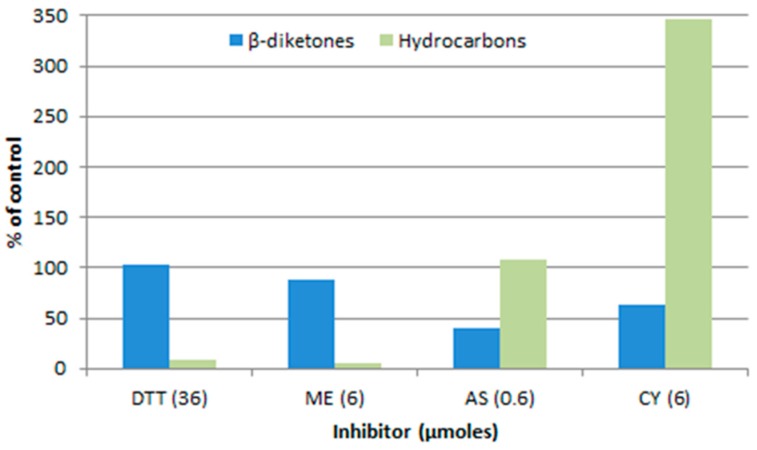
Preincubation with inhibitors affects the amount of label incorporated into the β-diketones and hydrocarbons of *cer-u.69* spike wax from [2-^14^C]-acetate [37]. DTT, 1,4-dithiothreitol; ME, 2-mercaptoethanol; AS, sodium arsenite; CY, potassium cyanide.

Another way to address the question as to the nature of the “KCS” extender, is to compare the sensitivities of the DKS polyketide and KCS pathways in barley spikes, to inhibitors. Figure 7 compares the effects of inhibitors on the β-diketones to that on the alkanes, in both of which the 31 carbon homolog dominates. The marked differences infer that the elongation steps beyond C_18_ are not carried out by the same set of enzymes, but do not identify the sensitive component(s) thereof [37].

Additional analyses of the aliphatics dominated by even chain lengths [58] revealed that 2-mercaptoethanol blocks their elongation at the C_20_ to C_22_ step, which is potentially the reason for the inhibition of alkane synthesis shown in Figure 7. A similar block was also identified for sodium arsenite, which is known to efficaciously inhibit the plastid localized KASII, and therefore, potentially able to inhibit the related β-ketoacyl-CoA synthase participating in the C_20_ to C_22_ extension. Thus, while these early results, as those of the effect of *cer* mutants on chain length distributions discussed above, suggest that different enzymes or complexes of carry out the “KCS” and KCS extensions, the question is still open. 

The possibility that DKS carries out the requisite extensions would appear to contradict its classification as a type III PKS. These enzymes use CoA substrates and carry out sequential reactions, that is, without intervening ones [1,3]. Assuming the same will be true for DKS, the starter substrate is a 3-oxo-acyl-CoA as illustrated in Figure 4. But while type III PKSs exhibit promiscuous substrate specificity, especially in vitro, can they do without a CoA in vivo? Secondly, can three intervening reactions to remove the 3-oxo group take place between each of the up to six extensions? If true, then the wild type β-diketone distributions in the 26 *Cer* loci whose mutants have reduced amounts of β-diketones, are accounted for, as are the marked differences in sensitivity to inhibitors to the KCS doing the elongations for the ubiquitous aliphatics. But this unexpected attribute would make DKS a very unusual type III PKS. 

### 2.11. The Third Type of Polyketide in Waxes, the Alkylresorcinols

**Figure 8:**
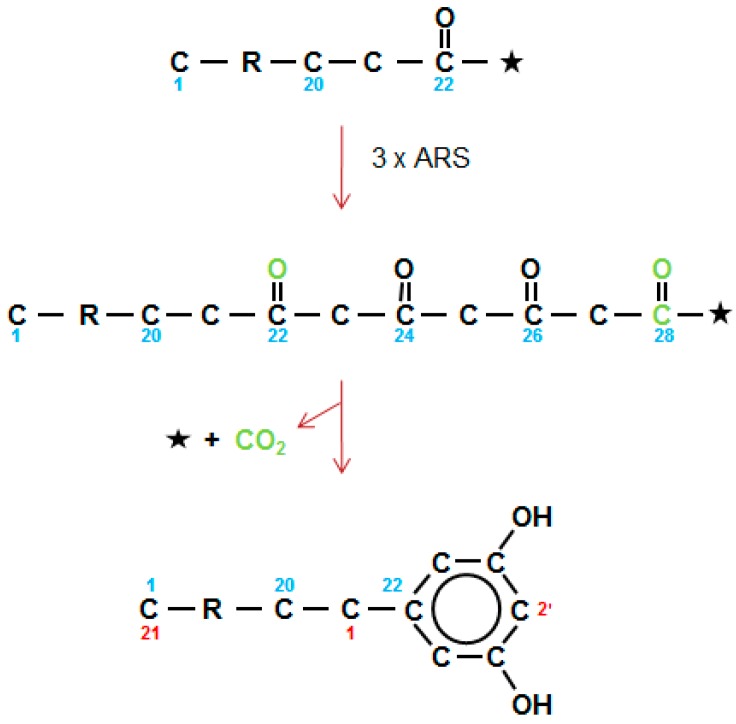
Type III alkylresorcinol synthases carry out three sequential elongations (3 × ARS) to give a theoretical tetraketide which then undergoes an aldol condensation, with the release of CoA (*) and CO_2_ (green) forming the 1,3-dihydroxybenzene ring. The 28 carbon tetraketide gives an AR with an alkyl chain of 21. Alkyl side chains range for 13–27 carbons. Numbering in direction of synthesis shown in blue, and as required for IUPAC nomenclature in red.

Already in 1974, Briggs [59] observed that the sum of the carbons in the alkyl chain plus the benzene ring of the ARs are similar to those of the alkanes present in the wax collected from the testa of barley grains. Four major homologs were identified, as well as a minor series with the potential to be branched ARs. More recently, homologous series of both ARs and methylARs (MARs), in approximately equal amounts, with alkyl chains of 19–29 carbons, have been documented in wheat flag leaf and peduncle waxes [19,60]. A similar AR series occurs in rye cuticular wax, but not in the epicuticular wax [61]. Quinoa grains have recently been shown to have a very complex mixture of ARs, including MARs, branched chain, unsaturated and even chain members [62]. The first AR synthase (ARS) genes (which are type III PKS-like) in plants, were cloned from sorghum and rice [20]. They included *ARS1* and *ARS2* from sorghum, plus three from rice. Among the acyl-CoA substrates used by ARS1 and ARS2 were C_16_ acyl-CoA chains with three double bonds that formed the C_22_ 1,3,5,7-tetraketide precursor of sorgoleone. Given an analogous pathway for the ARs in barley and wheat wax, then C_14–30_ saturated CoAs are used to form C_20–36_ tetraketides, which then undergo an aldol C_2_ → C_7_ condensation, with elimination of a CO_2_ (Figure 8), as carried out by ARS1 and ARS2, and also by stilbene synthases (STSs). For the MARs, one of the three condensations giving rise to the tetraketide, presumably uses methylmalonyl-CoA as an extender, instead of malonyl-CoA. In quinoa, this is the second of the three ARS extensions, as nuclear magnetic resonance located the methyl group in position 2′ of the resorcinol ring [62]. 

## 3. Conclusions

With the cloning of the *Cer-cqu* gene cluster, a new polyketide pathway that leads to major components of epicuticular waxes, has been established. Many interesting questions need to be addressed to fill in the details. Twelve of these follow:

(1) Are there *Cer-cqu* gene clusters in other species besides barley and wheat, for example, Eucalyptus, a dicot?
(2) What is the contribution of each member of the *Cer-cqu* cluster in wheat to synthesis of the polyketide aliphatics?
(3) What is the substrate for CER-Q?
(4) What is(are) the subcellular localizations of CER-Q, CER-C/DKS, and CER-U? If occurring in different compartments how are the substrates/products transferred from one to the other?
(5) How are the polyketide aliphatics transported to the cuticle surface?
(6) Does CER-C/DKS carry out additional polyketide partial reactions besides substrate recognition and condensation, such as cleavage of CoA from the final elongated carbon skeleton and its decarboxylation?
(7) In which direction are the carbon skeletons of the β-diketones synthesized in additional species, especially one of those with 2,4-oxo groups, for example, vanilla?
(8) How many condensations does CER-C/DKS carry out; that is, only the two initial ones resulting in retention of the two oxygens, or also all the subsequent six that are accompanied by the three accessory reactions removing the β-oxygens (Figure 4)?
(9) What genes determine the deduced thioesterase, decarboxylase, methylketone reductase, and ester synthase enzymes in the alkan-2-ol ester branch pathway?
(10) Why are *AtCER2* orthologs, that are required for the final KCS elongation steps of ubiquitous wax aliphatics [57], apparently not required for the final elongations in synthesis of the β-diketone aliphatics?
(11) What are the roles of the barley *Cer-a*, -*b*, -*x* and -*yl* loci products in eliminating almost all or all synthesis of polyketide wax aliphatics, and simultaneously modifying synthesis of ubiquitous wax aliphatics? Likewise, for the barley *Cer-YY* gene, a dominant inhibitor of spike polyketide wax aliphatics, whose mutants simultaneously change the spike ubiquitous wax aliphatics to resemble those found on wild type leaves [63].
(12) What genes regulate synthesis of polyketide aliphatics in addition to wheat *Iw1* and its potential homologues in other species, and how do they do so? Are they the same or different to those regulating the ubiquitous aliphatics?

## Figures and Tables

**Table 1:** Chain length and positions of oxo groups in β-diketones in plants and their location.

Plant	Location	Chain Length								Reference
		19	21	23	25	27	29	31	33	
Eucalyptus risdoni	Stem and leaf wax						12,14 *	14,16		[4]
Acacia podalyriaefolia + baileyana	Stem and leaf wax								16,18 *	[4]
Festuca glauca	Stem and leaf wax								12,14 *	[4]
Dianthus carophyllus	Stem and leaf wax						10,12 12,14	12,14 *	12,14 14,16	[4,5]
Hordeum vulgare	Spike, leaf sheath and internode wax						12,14 14,16	14,16 *	16,18	[5]
Triticum species	Spike, peduncle and flag leaf wax							14,16 *		[6]
Buxus sempervirens	Leaf wax						6,8	8,10 *	10,12	[7]
Rhodedendron baileyi	Leaf wax,						8,10	10,12 *		[8]
Rhodedendron racemosum + hemitrichotum	Leaf wax						8,10	14,16 *		[8]
Hosta “Krossa regal”	Leaf wax						10,12	10,12 ^◊^		[9]
Helianthus annus ^1^	Pollen coats	4,6	4,6 6,8 ^◊^	4,6 ^◊^ 6,8	4,6 ^◊^ 6,8 ^◊^ 10,12	4,6 6,8 10,12	6,8 10,12	10,12	10,12	[10]
Sphagnum section Acutifolia	Subfossil roots and leaflets				2,4	2,4 ^◊^	2,4 ^◊^	2,4		[11]
Vanilla fragrans + tahitensis ^2^	Oily gum in pods				2,4	2,4 *	2,4	2,4	2,4	[12]
Triticum aestivum	Flag leaf and peduncle waxes								2,4	[13]

^1^ In addition, also have 1-phenyl-1,3-C_16_*, C_18_ and C_20_ diones. ^2^ Contain cis double bond in direction of synthesis at 9–10. * Predominating or ^◊^ major β-diketone.

**Table 2:** Chain lengths of esterified alkan-2-ols in plant waxes and their location.

Plant	Location	β-DKs ^1^	Chain Length ^2^						Reference
			7	9	11	13	15	17	
Eucalyptus risdoni	Stem and leaf	present		xx	xx	xx	x		[14]
Eucalyptus globulus	Stem and leaf	present			xx	x	xx	x	[14]
Hordeum vulgare	See Table 3	present			x	xx	xxx	x	[15,16]
Sorghum bicolor	Seedling leaf	absent		x					[17]
Agropyron sp	Whole flowering plants	present				x	xx		[18]
Triticum aestivum	See Table 3	present	xx	x	x	xx	xxx	x	[19]

^1^ β-DKs, β-diketones; ^2^ x, xx and xxx denote increasing relative amounts of specified chain length deduced from data given in specified references.

**Table 3:** Location of β-diketone aliphatics.

Organ	Cuticle Surface	Barley [23]	Wheat [24]	Rye ^1^	Rice [28]
Spikes, panicles		+	+	+	−
Peduncles, leaf sheaths, internodes	Upper Lower	+ −	+ −	+ +	− −
Flag leaf	Adaxial Abaxial	− −	− +	− +	− −
Vegetative leaves	Adaxial Abaxial	− −	− −	− +	− −

^1^ Deduced from blue color in many varieties which is determined by the *Wa* gene that is syntenic [25] to barley *Cer-cqu* [26] and wheat *W1* [27].

**Table 4:** Chain length distributions^1^ of wild type Bonus and *cer* mutant β-diketones^2^.

	C_29_	C_31_	C_33_
Bonus	0.39	95.97	3.64
32 mutants from 26 *Cer* loci ^1^	0.51 ± 0.32	95.44 ± 1.02	4.02 ± 1.11

^1^ Average ± SD. ^2^ β-diketones analyzed as in [5]. The mutants were cer-c.3, c.63, d.5, e.8, f.9, k.39, o.28, r.19, v.49, w.48, h.13, i.16, n.26, n.93, n.97, n.624, n.985, t.46, u.69, u.699, yc.135, yd.139, yk.147, yt.938, zb.38, zc.65, zi.68, zr.260, zs.467, zt.479, zu.122, zx.100.

**Table 5:** ODs and alkane homolog distributions in spike waxes from Bonus and 12 *cer* mutants ^1^.

	OD_273_^2^	Homolog Carbon Number			Alkanes
		21 + 23 + 25	27 + 29	31 + 33	% of HC ^3^
Bonus	0.72	2.0	16.8	76.2	95.0
*b.64*	0.03	5.4	23.2	68.2	96.8
*a.6*	0.05	30.4	29.3	12.6	72.3
*a.12*	0.05	36.9	32.0	12.6	81.5
*a.33*	0.04	36.3	27.3	8.4	72.0
*yl.187*	0.19	43.1	28.8	9.7	81.6
*b.4*	0.08	4.8	51.8	39.8	96.4
*b.66*	0.05	12.5	40.0	40.8	93.3
*b.79*	0.05	6.5	43.0	44.5	94.0
*x.60*	0.12	23.1	26.5	23.7	73.3
*a.154*	0.51	14.8	30.0	44.2	89.0
*b.96*	0.40	4.1	20.9	71.3	96.3
*z.113*	0.11	13.2	15.0	63.5	91.7

^1^ Analyzed as in [15,56]. ^2^ β-diketones are not visible in thin layer chromatograms of the waxes with ODs ≤ 0.12. ^3^ HC, hydrocarbons.
